# Electronic Health Record Alerts to Improve Lipid Lowering After a Recent Myocardial Infarction

**DOI:** 10.1161/JAHA.125.047116

**Published:** 2026-06-09

**Authors:** Dean G. Karalis, Benjamin Richter, Scott Hessen, Patrick Moeller, Laney K. Jones

**Affiliations:** ^1^ Cardiology Consultants of Philadelphia Philadelphia PA USA; ^2^ Thomas Jefferson University Hospital Philadelphia PA USA; ^3^ Thomas Jefferson University Philadelphia PA USA; ^4^ Amgen Inc. Thousand Oaks CA USA

**Keywords:** guidelines, lipid‐lowering therapy, low‐density lipoprotein‐cholesterol, myocardial infarction, Secondary Prevention

## Abstract

**Background:**

This observational, quality initiative evaluated the impact of changing from a passive (dismissible) to an active (nondismissible without action) electronic health record alert on guideline‐recommended lipid‐lowering therapy (LLT) prescriptions in patients with recent myocardial infarction at risk for secondary events.

**Methods:**

We sequentially recruited a retrospective passive‐alert (February 2018–July 2019; n=733) and a prospective active‐alert (August 2020–January 2022; n=587) cohort of patients who triggered an alert to intensify LLT or order a low‐density lipoprotein‐cholesterol (LDL‐C) test if they had a recent myocardial infarction (within 12 months) and elevated (≥70 mg/dL) or missing LDL‐C. Prescribed LLTs and cumulative percentages of patients with missing or LDL‐C <70 and <55 mg/dL were assessed in 6‐month periods up to 24 months. Reasons for not intensifying LLTs were recorded with the active alert.

**Results:**

During 24 months, statin and high‐intensity statin use increased from 59% to 87% and from 39% to 69%, respectively, in the passive‐alert cohort. In the active‐alert cohort, statin and high‐intensity statin use were high and changed minimally (79% to 80% and 70% to 73%), but ezetimibe and proprotein convertase subtilisin/kexin type 9 inhibitor use increased from 12% to 35% and from 2% to 8% (odds ratio, 4.69 [95% CI, 3.22–6.96] and 4.07 [95% CI, 1.92–9.46]), respectively. LDL‐C testing and LDL‐C goal attainment improved in both cohorts. LDL‐C not current (41.7%) was the most common reason for not intensifying LLT.

**Conclusions:**

Continued efforts are needed to encourage guideline‐directed LLT intensification for patients with a recent myocardial infarction who are at risk of another cardiac event.

**Nonstandard Abbreviations and Acronyms**
ACC: American College of Cardiology
AHA: American Heart Association
CCP: Cardiology Consultants of Philadelphia
GOULD: Getting to an Improved Understanding of Low‐Density Lipoprotein‐Cholesterol and Dyslipidemia Management
LLT: lipid‐lowering therapy
PCSK9: proprotein convertase subtilisin/kexin type 9

**Clinical Perspective**

**What Is New?**

- Alerts embedded in electronic health records can be used to improve lipid‐lowering therapy intensification and low‐density lipoprotein cholesterol testing in patients with recent myocardial infarction who are not receiving guideline‐recommended lipid‐lowering therapy.

**What Are the Clinical Implications?**

- In this quality initiative comparing 2 sequential cohorts of 1651 (passive, dismissible alert) and 1524 (active, nondismissible alert without action) patients from a cardiology practice network, the continued use of statins and high‐intensity statins during 24 months and improved low‐density lipoprotein cholesterol goal attainment (<70 or <55 mg/dL) increased during both alert periods; nonstatin (ezetimibe and proprotein convertase subtilisin/kexin type 9 inhibitor) use and low‐density lipoprotein cholesterol testing improved further during the active alert compared with the passive alert.
- This exploratory analysis indicates that nondismissible best practice electronic health record alerts might improve guideline‐recommended lipid‐lowering therapy in patients with recent myocardial infarction at risk for secondary events and could be considered in clinical practice, especially when combined with additional strategies, such as patient and physician education, to address remaining gaps in low‐density lipoprotein cholesterol monitoring and lowering.

Intensive lipid‐lowering therapy (LLT) after a myocardial infarction (MI) can reduce the risk of recurrent atherosclerotic cardiovascular disease (ASCVD) events.^1^, ^2^, ^3^, ^4^ For patients with a recent MI, the 2018 American Heart Association (AHA)/American College of Cardiology (ACC) multisociety lipid management guidelines recommend a low‐density lipoprotein cholesterol (LDL‐C) threshold of 70 mg/dL for patients with very high‐risk ASCVD.^5^ The European Society of Cardiology (ESC) guidelines and the 2022 ACC Expert Consensus Decision Pathway on nonstatin LLTs recommend an LDL‐C threshold of 55 mg/dL and an LDL‐C reduction of ≥50% from baseline for these patients.^6^, ^7^ The 2018 guideline‐recommended LLTs for patients with very high‐risk ASCVD and LDL‐C above these thresholds included high‐intensity statins, with added ezetimibe and PCSK9 (proprotein convertase subtilisin/kexin type 9) inhibitors in a stepwise manner.^5^, ^6^, ^7^

Clinical decision support tools embedded within electronic health record (EHR) systems can alert clinicians in real‐time at the point of care by identifying high‐risk patients and facilitating patient‐specific orders based on recommended treatment guidelines.^8^, ^9^, ^10^ In 2017, Cardiology Consultants of Philadelphia (CCP) implemented a quality initiative, in which a passive EHR alert would trigger for patients with a recent MI and LDL‐C ≥70 mg/dL. The passive alert recommended to intensify LLT but could be dismissed without action. After 2 years, this passive EHR alert was changed to an active “hard‐stop” alert that could not be dismissed without action, which included the requirement to provide a reason for not prescribing guideline‐recommended LLT. We evaluated the impact of changing from a passive to an active alert for patients with a recent MI who were not receiving 2018 guideline‐recommended care.^5^ Our hypothesis was that the active alert would improve adoption of the 2018 management of cholesterol guideline recommendations by measuring LDL‐C and prescribing combination LLTs more often.

## METHODS

### Data Availability

Qualified researchers may request the data that support the findings of this study from Amgen clinical studies. Complete details are available at https://www.amgen.com/datasharing.

### Study Design

The quality improvement initiative was conducted within CCP, a practice network with 35 locations in the greater Philadelphia area that includes 96 physicians among cardiology subspecialties serving over 250 000 patients. This observational study compared 2 sequential cohorts of patients who triggered an EHR alert to their provider suggesting adoption of guideline‐recommended care, including LDL‐C testing and LLT prescriptions. Data were collected from February 2017 through October 2022. Only deidentified data were analyzed to protect study participants' confidentiality. The Office of Human Research Institutional Review Board for Thomas Jefferson University determined the study was exempt from institutional review board review and did not require informed consent from study participants.

### Participants

Eligible patients were identified from the CCP cardiac catheterization records at the first point‐of‐care (index) visit if they met criteria of very high cardiovascular risk (elevated or missing LDL‐C within 6 months and recent MI within 12 months of index visit) based on *International Classification of Diseases* (*ICD*) codes for coronary artery disease and MI (Table S1). Patients recruited during the active hard‐stop alert were prospectively identified (August 2020–January 2022) and followed for up to 24 months after index (Table S2). The control group was retrospectively identified during a previous passive‐alert period (February 2018–July 2019) using the same criteria as the active‐alert arm. All CCP cardiologists could receive an alert regarding their eligible patients, either passive or active, depending on the period of the patient's index visit. As part of the active alert, cardiologists were also required to attend a 1‐hour educational webinar at the start of the active‐alert period and complete a follow‐up survey at the end of the active‐alert period. No educational training or follow‐up surveys were provided as part of the passive alert.

### EHR Alert

Both alerts triggered for patients with similar eligibility criteria, ie, if the patient had a recent MI (within 12 months of index visit) and either elevated LDL‐C (≥70 mg/dL, not at goal based on the 2018 lipid management guidelines^5^) or missing LDL‐C within 6 months of index visit (LDL‐C not current) (Figure S1). If the patient's LDL‐C was <70 mg/dL within 6 months of or at the index visit, neither alert would trigger because the patient was considered to have met their LDL‐C goal. Patients who did not trigger either alert were excluded from this analysis.

With the active alert only, the physician was prompted to intensify LLT, provide a reason for not intensifying LLT, or order an LDL‐C test if not current, and schedule a follow‐up office visit. The initial prompt to intensify LLT included prescribing a statin if the patient was not already receiving one or to step up from a statin to a high‐intensity statin; if the patient was already receiving a high‐intensity statin, the initial prompt would be bypassed and the recommendation would be to add either ezetimibe or a PCSK9 inhibitor (Figure S1).

### Baseline Variables

Baseline characteristics recorded from the EHR and taken at index visit or, if missing, within 12 months before index, included patient demographics (age, sex, race, ethnicity, medical insurance type) and clinical characteristics (body mass index, systolic/diastolic blood pressure, comorbid conditions [hypertension, diabetes], smoking status). Preindex LDL‐C levels (or their absence) and prescribed LLTs were assessed during the 6‐month preindex period. Prescribed LLT medication was determined by having an active prescription for statins, ezetimibe, or PCSK9 inhibitors. Statin prescriptions were further classified as high intensity or not high intensity as defined in the 2018 guidelines.^5^

### Outcomes

The primary end point was prescribed LLT (or the continued use of each LLT) assessed in 6‐month postindex periods at 6, 12, 18, and 24 months, based on the presence or absence of active medication records for any LLTs during each 6‐month period. Secondary end points included numbers of LDL‐C tests with reported levels, as well as the last recorded LDL‐C levels within each period. Time to postindex LDL‐C testing and time to any LDL‐C level less than LDL‐C goals (≤70 mg/dL and ≤55 mg/dL) were evaluated. Cumulative percentages of patients with any postindex LDL‐C status were calculated using Kaplan–Meier methodology for 6‐, 12‐, 18‐, and 24‐month benchmarks. Frequencies of physician‐reported reasons for not prescribing guideline‐recommended intensifying LLTs and survey responses were also evaluated.

### Analyses

All characteristics and end points were reported descriptively before and after index for both cohorts, either as medians and interquartile ranges (IQRs) or frequencies (percentages or cumulative percentages). Mann–Whitney and χ^2^ tests were used when comparing medians and percentages, respectively, between cohorts. Binomial logistic regressions were performed to determine significant predictors of prescription of each LLT type immediately after index. Patients were not excluded from a medication use model if they previously received a LLT medication, because removal of the LLT type was an option for providers and the intended outcome was the continued use of each LLT after receiving the alert, not solely prescription in patients with no history of prescription for a medication. A multivariable Cox proportional hazards model compared relationships between prescribed LLT and other potential predictors with LDL‐C follow‐up testing or LDL‐C goal attainment. Analyses were performed using R (R Foundation for Statistical Computing), including statistical (version 4.4.3) and survival (version 3.8–3) packages.

## RESULTS

### Baseline Characteristics

During the passive‐alert period, 1651 patients had a qualifying MI; of these, 733 (44%) triggered the alert. During the active‐alert period, 1524 patients had a qualifying MI and 587 (39%) triggered the alert. The passive‐ and active‐alert cohorts had similar median ages (67 and 65 years, respectively), were predominantly men (66% and 61%) and of White race (84% and 79%), and had similar percentages of current smokers or patients with comorbidities (diabetes or hypertension) (TableTable). More patients were covered by Medicare in the passive‐alert cohort compared with the active‐alert cohort.

**Table 1:** Preindex Characteristics

	No. (%)		*P* value*
	Passive‐alert cohort (n=733)	Active‐alert cohort (n=587)	
Age, median (IQR), y†	67.0 (58.0–76.0)	65.0 (57.0–76.0)	0.152
Sex			
Female	247 (34)	230 (39)	0.045
Male	486 (66)	357 (61)	
Race			
Black	92 (13)	75 (13)	<0.001
White	613 (84)	461 (79)	
Other‡	28 (4)	51 (9)	
Hispanic or Latino ethnicity	9 (1)	18 (3)	0.032
Insurance			
Medicare	321 (44)	201 (34)	<0.001
Medicaid	30 (4)	53 (9)	
Commercial§	382 (52)	333 (57)	
Body mass index, median (IQR)‖ ^,^ #	29.2 (25.7–33.7)	29.1 (25.4–33.5)	0.350
Systolic blood pressure, median (IQR), mm Hg#	124 (114–132)	126 (118–134)	0.004
Diastolic blood pressure, median (IQR), mm Hg#	74.0 (68.0–80.0)	76.0 (70.0–80.0)	0.008
Hypertension**	524 (71)	423 (72)	0.866
Diabetes††	178 (24)	153 (26)	0.498
Current smoker	80 (11)	61 (10)	0.916
LDL‐C level,‡‡ median (IQR), mg/dL	101 (83–124)	93 (78–117)	<0.001
LDL‐C level categories‡‡			
Missing LDL‐C level	290 (40)	183 (31)	0.001
70–99 mg/dL§§	211	219	
≥ 100 mg/dL§§	232	185	
LLT type‡‡ ^,^ ‖‖			
Any statin##	430 (59)	465 (79)	<0.001
High‐intensity statin##	287 (39)	409 (70)	<0.001
Ezetimibe	27 (4)	69 (12)	<0.001
PCSK9 inhibitor***	2 (0.3)	12 (2)	0.004
Number of LLTs‡‡			
0	297 (41)	109 (19)	<0.001
1	415 (57)	414 (71)	
≥2	21 (3)	64 (11)	

EHR indicates electronic health record; *ICD‐10‐CM*, *International Classification of Diseases, Tenth Revision, Clinical Modification*; IQR, interquartile range; LDL‐C, low‐density lipoprotein cholesterol; LLT, lipid‐lowering therapy; and PCSK9, proprotein convertase subtilisin/kexin type 9.

* χ2 test used when comparing frequencies and Mann–Whitney when comparing medians.

^†^ Calculated as whole year, rounded down, at index visit using birthdate in EHR.

^‡^ Other included all races other than White or Black/African American, missing, and refused to answer.

^§^ Commercial included any insurance provider listed in the EHR with a text that did not match Medicare, Medicaid, or any known Medicaid plan.

^‖^ Calculated as weight in kilograms divided by height in meter squared.

^#^ Calculated using values recorded in the EHR at index visit or, if missing, values recorded at the most recent visit within the previous 12 months before the index visit.

** Defined by the presence of *ICD‐10‐CM* codes I1x for any hypertensive disease in the patient's EHR and diagnosis files.

^††^ Defined by the presence of *ICD‐10‐CM* codes E08, E09, E11, E12, or E13 for type 2 diabetes in the patient's EHR and diagnosis files.

^‡‡^ Within 6 months before or on index visit.

^§§^ For patients with values.

^‖‖^ Included patients receiving single or combination therapies; some patients may be included in several categories.

^##^ Including atorvastatin, fluvastatin, lovastatin, pitavastatin, pravastatin, rosuvastatin, and simvastatin. Further classified as high‐intensity or not based on dosages defined in the 2018 American Heart Association/American College of Cardiology Multisociety Guideline on the Management of Blood Cholesterol.^5^

*** Including alirocumab and evolocumab.

Fewer patients had missing 6‐month preindex LDL‐C levels in the active‐alert cohort (31%) compared with the passive‐alert cohort (40%; *P*=0.001). For those with a recorded LDL‐C level, the median was significantly lower in the active‐ versus passive‐alert cohort (93 mg/dL versus 101 mg/dL, *P*<0.001). Preindex usage of any statin, high‐intensity statin, ezetimibe, or PCSK9 inhibitor was significantly greater in the active‐ versus the passive‐alert cohort; high‐intensity statin monotherapy was prescribed frequently (Table S3).

### Prescription of LLTs

**Figure 1:**
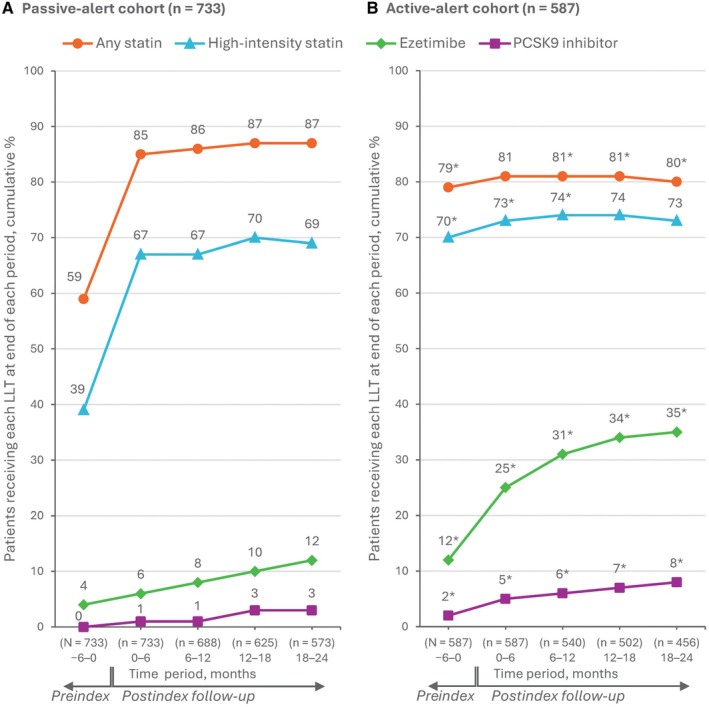
Impact of intervention on postindex intensification of guideline‐recommended LLT. **A**, Passive‐alert cohort (n=733). **B**, Active‐alert cohort (n=587). Orange dots and lines, blue triangles and lines, green diamonds and lines, and purple squares and lines, represent any statin, high‐intensity statin, ezetimibe, and PCSK9 inhibitor, respectively. LLT indicates lipid‐lowering therapy; and PCSK9, proprotein convertase subtilisin/kexin type 9. *P<0.05 comparing active‐alert cohort with passive‐alert cohort (χ^2^ test).

In the passive‐alert cohort, statin use increased from 59% preindex to 85% to 86% throughout follow‐up, and high‐intensity statin use increased from 39% to 67% to 70% (Figure 1A); few patients received ezetimibe or PCSK9 inhibitors preindex or during follow‐up in this cohort. During the active‐alert period, statin and high‐intensity statin use remained elevated, with little change during follow‐up; in contrast, ezetimibe use increased from 12% to 35% and PCSK9 inhibitor use from 2% to 8% (Figure 1B).

By 24 months, the percentage of patients in the passive‐alert cohort who were not prescribed any LLT decreased markedly from 41% to 11%, while use of single (from 57%–77%) and combination (from 3–12%) LLTs increased (Figure S2). In the active‐alert cohort, the use of combination LLTs increased from 11% to 35%, including combination of a statin with ezetimibe or a PCSK9 inhibitor in 32.9% (150/456) and 5.4% (25/456) of patients (Table S3; Figure S3). Patients with preindex LDL‐C ≥100 mg/dL had the greatest use of combination therapies (9.1% in passive‐ and 31.9% in active‐alert cohorts) (Figure S4). Furthermore, having a preindex LDL‐C test and recorded level increased the likelihood of being prescribed an LLT, especially combination LLTs.

Binomial logistic regressions identified significant predictors of LLT prescription (Table S4). Patients from the active‐alert cohort were 4.69 and 4.07 times more likely to have a postindex prescription for ezetimibe (95% CI, 3.22–6.96) or a PCSK9 inhibitor (95% CI, 1.92–9.46) compared with patients from the passive‐alert cohort after adjustment for other covariates. Patients with preindex LDL‐C ≥100 mg/dL had adjusted odds ratios (ORs) of 2.86 (95% CI, 1.83–4.54) and 3.57 (95% CI, 1.53–9.37) to receive postindex ezetimibe or PCSK9 inhibitor, respectively, compared with those missing preindex LDL‐C. Adding insurance coverage type to the models had no impact on the findings. The strongest predictor for prescription of a specific LLT postindex was having a preindex prescription for that LLT.

### LDL‐C Monitoring and Goal Attainment

**Figure 2:**
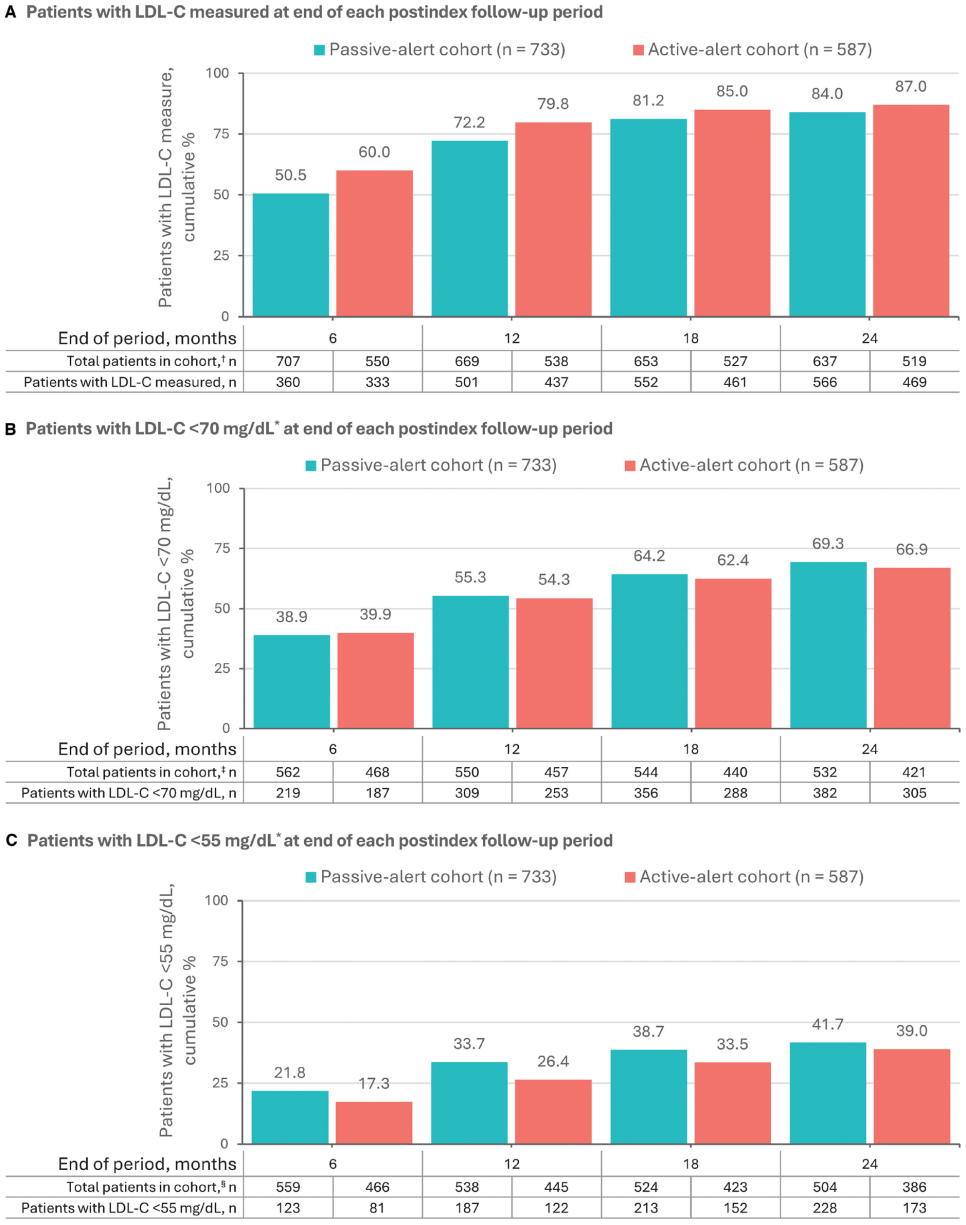
Impact of intervention on postindex LDL‐C testing and goal attainment at the end of each postindex follow‐up period.* **A**, LDL‐C measurements. **B**, Patients with LDL‐C <70 mg/dL. **C**, Patients with LDL‐C <55 mg/dL. EHR indicates electronic health record; and LDL‐C, low‐density lipoprotein cholesterol. *Evaluated only in patients with LDL‐C values recorded in the EHR using a survival analysis. ^†^Total number of patients in the cohort followed to the end of the study period or to having a recorded LDL‐C value in the EHR before the end of the study period. ^‡^Total number of patients in the cohort followed to the end of the study period or to having a recorded LDL‐C <70 mg/dL in the EHR before the end of the study period. ^§^Total number of patients in the cohort followed to the end of the study period or to having a recorded LDL‐C <55 mg/dL in the EHR before the end of the study period.

Frequencies of missing LDL‐C were significantly lower in the active‐alert cohort compared with the passive‐alert cohort at 6 months (40.4% [237 of 587] versus 49.8% [365 of 733]; *P*<0.001); they continued to decrease for up to 24 months and remained slightly lower in the active‐ versus the passive‐alert cohort (Table S5). In parallel, cumulative percentages of patients with LDL‐C levels on record increased for both cohorts (Figure 2A), with a significant difference in favor of the active‐alert cohort (*P*<0.001; log‐rank test; Figure S5).

For patients with measured postindex LDL‐C, cumulative percentages of LDL‐C goal attainment of <70 mg/dL and <55 mg/dL increased over time (Figure 2B and 2C) but did not differ significantly between cohorts (passive alert, *P*=0.4 versus active alert, *P*=0.07; log‐rank test; Figure S5). Medians of last recorded LDL‐C measures ≤6 months postindex did not differ significantly between cohorts (passive alert, 68.0 [IQR, 56.0–86.0] versus active alert, 64.5 [IQR, 51.0–84.3]; *P*=0.086) but were <70 mg/dL through 24 months of follow‐up (Table S5).

**Figure 3:**
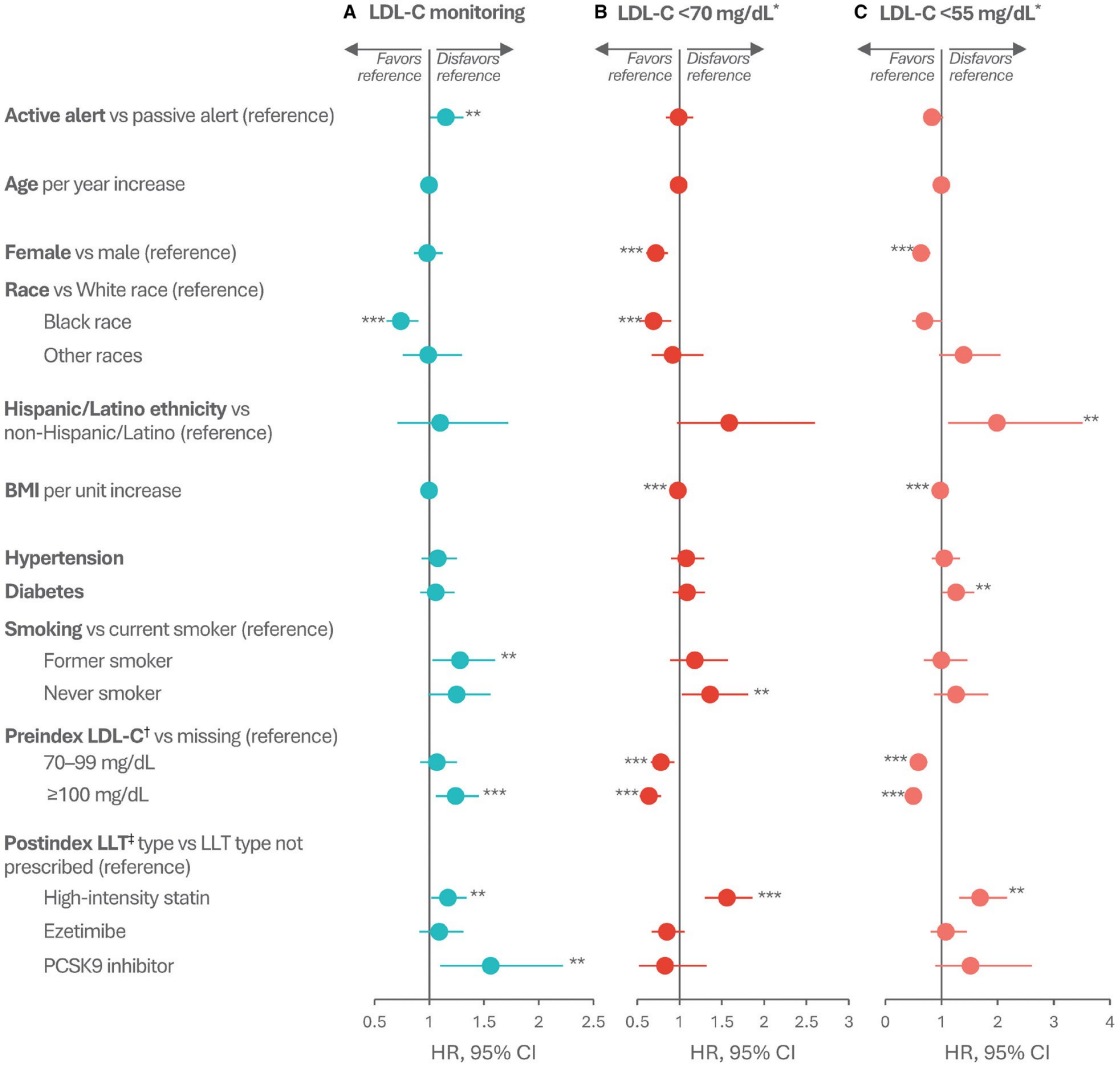
Predictors of improved LDL‐C testing and goal attainment.* **A**, LDL‐C monitoring. **B**, LDL‐C of <70 mg/dL. **C**, LDL‐C<55 mg/dL. BMI indicates body mass index; HR, hazard ratio; LDL‐C, low‐density lipoprotein cholesterol; LLT, lipid‐lowering therapy; and PCSK9, proprotein convertase subtilisin/kexin type 9. **P<0.05; ***P<0.01. *For patients with values. ^†^Recorded 6 months before or on index visit. ^‡^Prescribed during initial 0 to 6 months postindex follow‐up period.

Characteristics substantially increasing the likelihood of LDL‐C monitoring during follow‐up were initial postindex prescription of high‐intensity statins or PCSK9 inhibitors, former smoking status, and preindex LDL‐C ≥100 mg/dL (Figure 3A, Table S6). However, patients of Black race were less likely to have increased LDL‐C testing compared with patients of White race. Patients of female (versus male) sex, Black (versus White) race, increasing BMI, and preindex LDL‐C ≥100 mg/dL (versus missing) were less likely to achieve an LDL‐C goal of <70 mg/dL compared with their respective reference groups (Figure 3B). Similar results were reported for the stricter LDL‐C goal of <55 mg/dL (Figure 3C). Postindex prescription of high‐intensity statins significantly increased the likelihood of attaining both LDL‐C goals. The number of LLTs prescribed did not impact LDL‐C goal attainment in either cohort (Figures S6 and S7). Adding insurance coverage type to the models had no impact on the findings.

### Physician‐Reported Reasons for Not Prescribing LLTs

**Figure 4:**
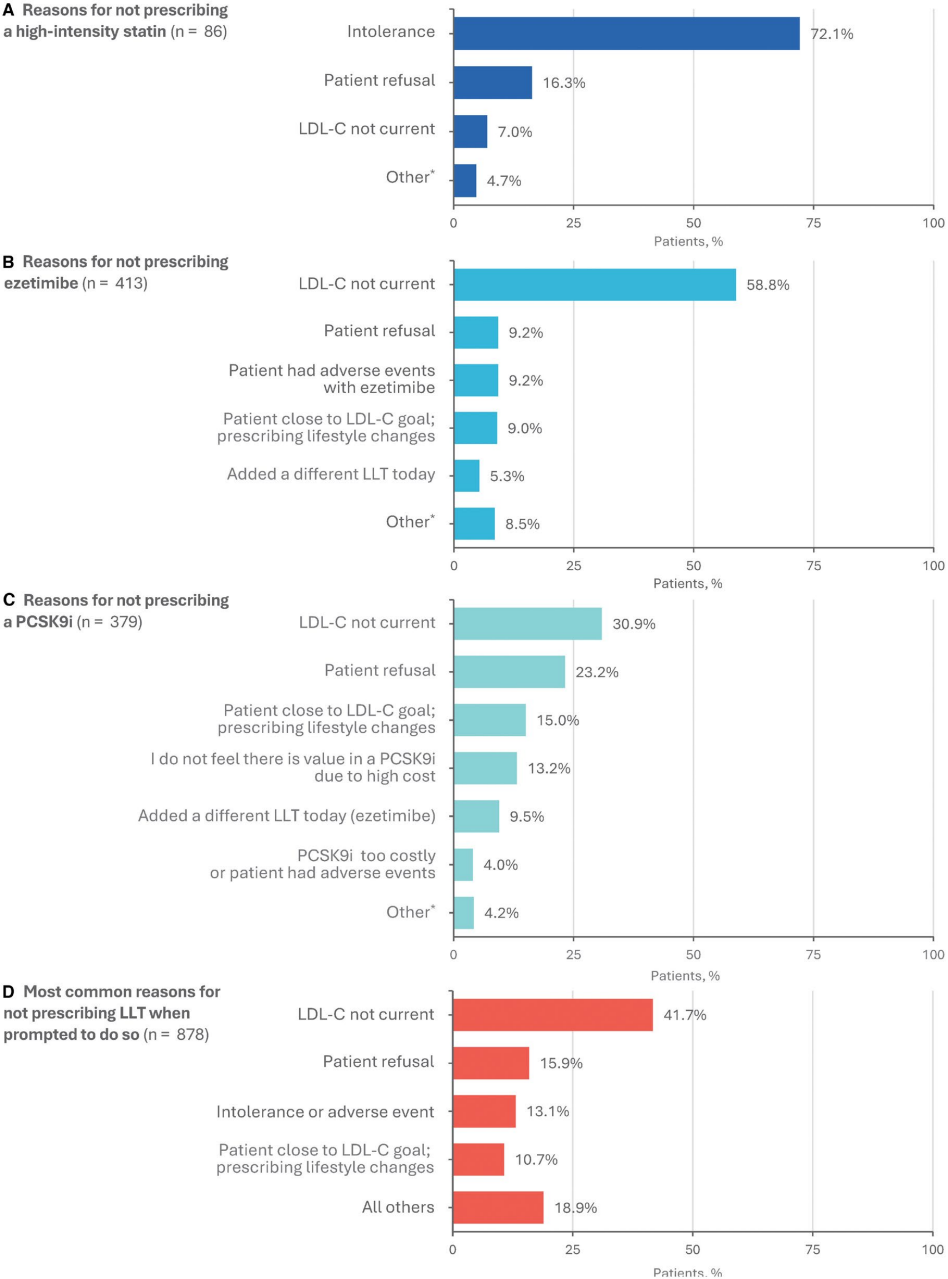
Reasons for active alert dismissal. **A**, Not to prescribe a high‐intensity statin (n=86). **B**, Not to prescribe ezetimibe (n=413). **C**, Not to prescribe a PCSK9i (n=379). **D**, Not to prescribe an LLT (n=878). LDL‐C indicates low‐density lipoprotein cholesterol; LLT, lipid‐lowering therapy; and PCSK9i, proprotein convertase subtilisin/kexin type 9 inhibitor. *Reasons given by<5 patients were combined into the “Other” category.

The most common reasons physicians gave for not prescribing high‐intensity statins were intolerance (62 of 86, 72.1%) and patient refusal (14 of 86, 16.3%) (Figure 4A). Reasons given for not prescribing ezetimibe were LDL‐C not current (243 of 413, 58.8%) and patient refusal or adverse events (38 of 413, 9.2%) (Figure 4B), and reasons for not prescribing a PCSK9 inhibitor were LDL‐C not current (117 of 319, 30.9%) and patient refusal (88 of 379, 23.2%) (Figure 4C). Overall, the most common reasons for not prescribing any LLT were LDL‐C not current (41.7%), patient refusal (15.9%), and intolerance (13.1%) (Figure 4D).

Of 96 CCP physicians, 88 (92%) completed the postintervention survey. From these responses, 95% (84 of 88) felt the preintervention training was effective, and most believed the intervention was effective in improving LLT management for patients with a recent MI (very effective, 23%; somewhat effective 58%) (Table S7).

## DISCUSSION

Findings from this observational sequential cohort study suggest that the passive EHR alert was associated with a rapid increase of statin prescriptions, including high‐intensity statins, but a minimal increase of prescriptions for nonstatins over 24 months. The change from a passive to an active alert resulted in an additional substantial increase in prescriptions for high‐intensity statins, ezetimibe, and PCSK9 inhibitors. LDL‐C testing and achieving levels <70 and <55 mg/dL increased with both alerts, with the active alert associated with most effective improvement in LDL‐C testing and both alerts associated with similar improvement in LDL‐C goal attainment. More than half of patients achieved an LDL‐C <70 mg/dL within 6 months with both the passive and active alert implementation (median LDL‐C of 68.0 mg/dL and 64.5 mg/dL, respectively). In a recent trial of high‐risk patients whose LDL‐C was >70 mg/dL, clinicians were randomized to receive a passive EHR alert that could be dismissed without action or no alert.^9^ The percentage of patients in whom LLT was intensified was numerically greater but not statistically significant between the groups.^9^ However, clinicians who did not dismiss the alert had a significant increase in LLT intensification of more than 2‐fold. Our findings, along with others, suggest that an active alert that cannot be dismissed without action or a reason is more effective in improving LLT intensification than a passive alert that can be dismissed.

Greater percentages of patients who triggered the active alert in our study were taking statin or high‐intensity statin medication than reported in contemporary observational studies of statin use in patients with ASCVD.^11^, ^12^, ^13^, ^14^, ^15^, ^16^, ^17^ Since both cohorts were treated by the same physicians and recruited sequentially, physicians may have benefited from the passive alert on how to manage LLT in high‐risk patients by the time the active alert was instituted. Lower statin usage in the active‐ compared with the passive‐alert cohort could be attributable to a higher prevalence of statin‐intolerant patients in the active‐alert cohort. The best predictor (by several orders of magnitude) of being prescribed an LLT was to have been prescribed that same LLT in the preindex period, suggesting that most prescriptions were continuing prescriptions from the preindex period. Our findings are in contrast to observational studies that have demonstrated that adherence to statin therapy often declines in the months after a patient is discharged from the hospital following an acute MI. In a large cohort of Medicare patients who filled a high‐intensity statin prescription within 30 days of their MI discharge, only 59% maintained high adherence at 6 months, and only 42% did so at 2 years.^18^ Our study suggests that an EHR prompt may improve compliance with statin therapy.

Overall, women appeared less likely to be prescribed high‐intensity statins or ezetimibe than men, as previously reported.^13^, ^16^, ^19^ This, in part, may be related to a higher prevalence of statin intolerance in women compared with men and to an underestimation of risk in women on the parts of both the clinician and the patient.^20^ In addition, patients with elevated LDL‐C were more likely to be prescribed nonstatin LLTs, including ezetimibe for LDL‐C >70 mg/dL and PCSK9 inhibitors for LDL‐C ≥100 mg/dL. This suggests that sex and baseline LDL‐C might be driving the likelihood of being prescribed intensified LLTs, and baseline LDL‐C would determine which therapy would be prescribed.

LDL‐C monitoring improved from baseline in the passive‐alert cohort and further improved in the active‐alert cohort. The rate of missing LDL‐C measures remained low over 24 months in both cohorts, indicating that the testing rate remained high and demonstrated persistence in testing throughout the study. When adjusting for all covariates, patients from the active‐alert cohort were more likely to have their LDL‐C measured compared with the passive‐alert cohort, but patients of Black race were less likely to have their LDL‐C measured and achieve an LDL‐C <70 mg/dL than those of White race. Lower statin use in patients of Black or Hispanic versus White race (23.8% or 23.9% versus 37.6%) was previously reported in a representative US population,^14^ a retrospective claims analysis,^17^ and during the COVID‐19 pandemic.^21^ Disparities in access to specialty care, socioeconomic barriers, and differences in patient beliefs and perceptions about statins may contribute to undertreatment in Black patients.^22^ Effective strategies for improving lipid management in high‐risk female and Black patients include a multilevel approach targeting providers, patients, and health care systems, with an emphasis on regular LDL‐C monitoring, patient education, and expanded access to combination LLTs.

In the subset of patients with measured postindex LDL‐C, goal attainment of <70 mg/dL and <55 mg/dL was similar for both cohorts. One potential reason for the active alert not improving LDL‐C goal attainment over the passive alert may be related to the fact that more patients in the active‐alert cohort were statin intolerant. Approximately 20% of the active‐alert cohort were prescribed no statin therapy. Another reason may be that LDL‐C was measured more frequently in the active‐alert cohort, therefore identifying more patients not achieving desired LDL‐C levels. In addition, ezetimibe lowers LDL‐C modestly and may not have been potent enough to achieve patient‐specific LDL‐C goals, especially at the more aggressive target of <55 mg/dL. Increased use of PCSK9 inhibitors would likely have led to more patients achieving their LDL‐C goals. Although the use of PCSK9 inhibitors increased in the active‐alert cohort, the overall use, including in combination with a statin, remained low.

However, LDL‐C goal attainment rates were high for both cohorts. The cumulative percentage of patients achieving LDL‐C <70 mg/dL was close to 70% at 24 months, in line with high‐intensity statin use rates of 69% to 73%. In comparison, in the GOULD (Getting to an Improved Understanding of Low‐Density Lipoprotein‐Cholesterol and Dyslipidemia Management) registry, 49.5% and 42.7% of patients with ASCVD who had baseline LDL‐C measures of 70 to 99 mg/dL and ≥100 mg/dL were taking high‐intensity statins at 2 years and only 33.9% and 21.0% achieved the LDL‐C goal of <70 mg/dL, respectively.^23^ In a US retrospective cohort study, 33.5% of patients with ASCVD and baseline LDL‐C 70 to 99 mg/dL and 21.0% of those with baseline LDL‐C 100 to 129 mg/dL achieved the LDL‐C goal of <70 mg/dL at 1 year of follow‐up.^15^ Greater rates of LDL‐C goal attainment in our study might be attributable to patients being treated exclusively by cardiologists compared with primary care or family physicians in other studies; it may also be attributable to the long‐term effects of our EHR best‐practice active alert itself, which took place after an initial passive alert and also included an educational component for clinicians.

### Limitations

This study was conducted within a single cardiology practice, and findings may not be generalizable to other cardiology or general practices. Randomized trials are considered the gold standard to establish causal effects, while nonrandomized, observational trials are more vulnerable to systemic differences between groups. Because our study was observational and not randomized, causal inference is limited, and our findings are exploratory. A randomized controlled design would have been better suited to definitively assess the causal effect of each alert. The observational, nonrandomized design of our study may have led to unequal distribution of known and unknown characteristics between our 2 cohorts, which may affect the validity of our results. For example, not all racial and ethnic patient groups were represented at a similar distribution to those of the general US population. Both cohorts were treated by the same physicians and recruited sequentially during different periods, and our physicians may have benefited from the passive‐alert period on how to manage LLT in high‐risk patients during the active‐alert period. This is supported by the finding that the active‐ and passive‐alert cohorts differed significantly in pre‐ndex and 6‐month LLT prescriptions, as well as baseline LDL‐C levels. Therefore, experience with the passive alert may have led to improved monitoring of LDL‐C and increased use of nonstatin therapies during the active‐alert period, which may make it difficult to attribute these benefits to the active alert. In addition, both cohorts were recruited during a time when the 2018 AHA/ACC multisociety lipid management guidelines were available and recommended an LDL‐C threshold of <70 mg/dL for intensifying LDL‐C–lowering therapies,^5^ but the 2022 ACC Expert Consensus Decision Pathway on nonstatin LLTs, which recommends an LDL‐C threshold of <55 mg/dL for add‐on LDL‐C–lowering therapies, had not yet been published.^7^ This may explain the low number of patients who achieved LDL‐C <55 mg/dL in our study. However, during the active‐alert period, the results of the outcome studies of the 2 available PCSK9 inhibitors were published,^3^, ^4^ which may also have influenced the increased use of PCSK9 inhibitors seen in our active‐alert cohort. In addition, patient characteristics as well as physician behaviors and prescription practices may change over time, potentially explaining some of the differences between the cohorts. Although the active‐alert cohort was recruited during the COVID‐19 pandemic, a preliminary sensitivity analysis indicated that any unobserved effect of pandemic‐era care practices would have needed to be large in comparison with the effect of any existing significant variables in our model to overwhelm the observed effect of alert type. Last, we could not assess whether the reason for less statin use in the active‐alert cohort was related to statin intolerance because data on statin intolerance were not collected at an individual patient level.

## CONCLUSIONS

This observational sequential cohort study suggests that both passive and active alerts can improve LDL‐C goal attainment primarily by increasing the use of statins, especially high‐intensity statins. In addition, the alerts either improved or maintained the continued prescriptions of statin therapy over 24 months. The active alert was associated with improving further LDL‐C testing and use of nonstatin therapies compared with the passive alert but not with improving LDL‐C goal attainment. The study also identified gaps in LDL‐C monitoring and lowering for high‐risk patients as well as subsets of patients who were less likely to have LDL‐C measured or treated to goal, such as Black and female patients. For patients with an MI within the past 12 months, opportunities to intensify LLTs and monitor LDL‐C were frequently missed despite the EHR alert. Nevertheless, both alerts were beneficial, demonstrating that EHR alerts should be considered in clinical practice. However, additional strategies complementing EHR hard‐stop best practice alerts need to be explored to overcome common reasons for not intensifying LLT, including greater use of nonstatin LLTs, better routine monitoring of LDL‐C and consideration of LDL‐C management as a quality measure, physician and patient education, and addressing adverse events and intolerance.

## Source of Funding

This study was funded by Amgen Inc., Thousand Oaks, CA. The funder had no role in the design and conduct of the study; collection, management, analysis, and interpretation of the data; preparation, review, or approval of the manuscript; and decision to submit the manuscript for publication.

## Disclosures

Dr Karalis has consulted for Amgen Inc., and Novartis and was on the speaker's bureau for Amgen Inc. Dr Jones is an employee of and holds stock in Amgen Inc. The remaining authors have no disclosures to report.

## Supporting information

Tables S1–S7
Figures S1–S7

STROBE Checklist
