# Supplementary material for: Electronic Health Record Alerts to Improve Lipid Lowering After a Recent Myocardial Infarction
Source: J Am Heart Assoc. 2026 Jun 9;15(12):e047116. doi: 10.1161/JAHA.125.047116 (PMC13323587; doi:10.1161/JAHA.125.047116)
Supplement: Supplementary file 1 — Tables S1–S7 Figures S1–S7 [file JAH3-15-e047116-s001.pdf]

SUPPLEMENTAL MATERIAL

Table S1. Diagnosis and procedure codes for triggering the EHR alert

Condition	Codes
MI-related procedure	*ICD-10-PCS:* 9345426, 9345526, 9345726, 9345826, 9345926, 9346026, 9346126
MI diagnosis for eligibility	*ICD-10-CM*: I21, I21.0, I21.01, I21.02, I21.09, I21.1, I21.11, I21.19, I21.2, I21.21, I21.29, I21.3, I21.4, I21.9, I22, I22.0, I22.1, I22.8, I22.9, I23, I23.0, I23.1, I23.2, I23.3, I23.4, I23.5, I23.6, I23.8
Takotsubo for ineligibility	*ICD-10-CM:* I51.81
Myocarditis for ineligibility	*ICD-10-CM:* I51.4

EHR, electronic health record; *ICD-10-CM*, *International Classification of Diseases, Tenth Revision, Clinical Modification*; *ICD-10-PCS*, *International Classification of Diseases, Tenth Revision, Procedure Coding System*; MI, myocardial infarction.

Table S2. Interventions

	Clinician education	Passive alert	Active alert
**Description**	1-hour webinar (on patient education for management of high-risk ASCVD) and brief follow-up survey (on perceptions of active EHR prompts)	EHR prompts that can be dismissed	Hard-stop EHR prompts that cannot be dismissed without action (either prescribing intensified LLT or selecting a reason for not intensifying LLT)
**Actors**	Clinicians		
**Action**	Clinicians were<br>• required to attend a 1-hour webinar<br>• invited to take a follow-up survey	Clinicians received an EHR prompt at point-of-care visit that they could either follow or dismiss	Clinicians received an EHR prompt at point-of-care visit that they could not dismiss without action
**Temporality**	• Educational webinar: at start of active-alert period<br>• Follow-up survey: at study completion	At index point-of-care visit for qualifying patients	At index point-of-care and follow-up visits for qualifying patients
**Frequency**	Once per clinician	Once for each qualifying ASCVD high-risk patient	Multiple times for each qualifying ASCVD high-risk patient while they qualify for the alert

ASCVD, atherosclerotic cardiovascular disease; EHR, electronic health record; LDL-C, low-density lipoprotein cholesterol; LLT, lipid-lowering therapy.

Table S3. Pre- and post-index LLT combinations prescribed

	N (%)		*P* value[*]
	**Passive-alert cohort**	**Active-alert cohort**	
Pre-index[†]	***N = 733***	***N = 587***	
No therapy	297 (41)	109 (19)	<.0001
Non-high-intensity statin only	134 (18)	50 (9)	<.0001
High-intensity statin only	275 (38)	354 (60)	<.0001
Ezetimibe only	6 (1)	7 (1)	.58
PCSK9 inhibitor only	0 (0)	3 (1)	.09
Non-high-intensity statin + ezetimibe	9 (1)	6 (1)	.80
High-intensity statin + ezetimibe	10 (1)	49 (8)	<.0001
Non-high-intensity statin + PCSK9 inhibitor	0 (0)	0 (0)	NA
High-intensity statin + PCSK9 inhibitor	0 (0)	2 (0)	.20
Ezetimibe + PCSK9 inhibitor	0 (0)	3 (1)	.09
Non-high-intensity statin + ezetimibe + PCSK9 inhibitor	0 (0)	0 (0)	NA
High-intensity statin + ezetimibe + PCSK9 inhibitor	2 (0)	4 (1)	.42
0–6 months post-index[‡]	***n = 733***	***n = 587***	
No therapy	105 (14)	95 (16)	.36
Non-high-intensity statin only	117 (16)	31 (5)	<.0001
High-intensity statin only	459 (63)	302 (51)	<.0001
Ezetimibe only	5 (1)	6 (1)	.55
PCSK9 inhibitor only	2 (0)	6 (1)	.15
Non-high-intensity statin + ezetimibe	13 (2)	12 (2)	.84
High-intensity statin + ezetimibe	25 (3)	113 (19)	<.0001
Non-high-intensity statin + PCSK9 inhibitor	2 (0)	1 (0)	.99
High-intensity statin + PCSK9 inhibitor	1 (0)	6 (1)	.05
Ezetimibe + PCSK9 inhibitor	1 (0)	4 (1)	.18
Non-high-intensity statin + ezetimibe + PCSK9 inhibitor	0 (0)	1 (0)	.45
High-intensity statin + ezetimibe + PCSK9 inhibitor	3 (0)	10 (2)	.02
6–12 months post-index[‡]	***n = 688***	***n = 540***	
No therapy	88 (13)	82 (15)	.24
Non-high-intensity statin only	107 (16)	27 (5)	<.0001
High-intensity statin only	430 (62)	251 (46)	<.0001
Ezetimibe only	5 (1)	8 (1)	.26
PCSK9 inhibitor only	2 (0)	9 (2)	.01
Non-high-intensity statin + ezetimibe	17 (2)	12 (2)	.85
High-intensity statin + ezetimibe	31 (5)	125 (23)	<.0001
Non-high-intensity statin + PCSK9 inhibitor	3 (0)	0 (0)	.26
High-intensity statin + PCSK9 inhibitor	2 (0)	6 (1)	.15
Ezetimibe + PCSK9 inhibitor	2 (0)	3 (1)	.66
Non-high-intensity statin + ezetimibe + PCSK9 inhibitor	0 (0)	2 (0)	.19
High-intensity statin + ezetimibe + PCSK9 inhibitor	1 (0)	15 (3)	<.0001

Table S3. Pre- and post-index LLTs prescribed *(Continued)*

	N (%)		*P* value*
	**Passive-alert cohort**	**Active-alert cohort**	
12–18 months post-index‡	***n = 628***	***n = 502***	
No therapy	72 (12)	80 (16)	.036
Non-high-intensity statin only	86 (14)	24 (5)	<.0001
High-intensity statin only	396 (63)	216 (43)	<.0001
Ezetimibe only	3 (0)	6 (1)	.20
PCSK9 inhibitor only	4 (1)	7 (1)	.23
Non-high-intensity statin + ezetimibe	19 (3)	9 (2)	.25
High-intensity statin + ezetimibe	32 (5)	132 (26)	<.0001
Non-high-intensity statin + PCSK9 inhibitor	3 (0)	1 (0)	.63
High-intensity statin + PCSK9 inhibitor	3 (0)	5 (1)	.48
Ezetimibe + PCSK9 inhibitor	2 (0)	4 (1)	.42
Non-high-intensity statin + ezetimibe + PCSK9 inhibitor	1 (0)	2 (0)	.59
High-intensity statin + ezetimibe + PCSK9 inhibitor	4 (1)	16 (3)	<.01
18–24 months post-index‡	***n = 573***	***n = 456***	
No therapy	61 (11)	71 (16)	.02
Non-high-intensity statin only	84 (15)	20 (4)	<.0001
High-intensity statin only	351 (61)	190 (42)	<.0001
Ezetimibe only	3 (1)	6 (1)	.20
PCSK9 inhibitor only	5 (1)	9 (2)	.18
Non-high-intensity statin + ezetimibe	20 (3)	10 (2)	.27
High-intensity statin + ezetimibe	38 (7)	121 (27)	<.0001
Non-high-intensity statin + PCSK9 inhibitor	3 (1)	0 (0)	.26
High-intensity statin + PCSK9 inhibitor	1 (0)	6 (1)	.05
Ezetimibe + PCSK9 inhibitor	3 (1)	4 (1)	.71
Non-high-intensity statin + ezetimibe + PCSK9 inhibitor	0 (0)	1 (0)	.44
High-intensity statin + ezetimibe + PCSK9 inhibitor	4 (1)	18 (4)	<.001

LLT, lipid-lowering therapy; PCSK9, proprotein convertase subtilisin/kexin type 9.

*Fisher's exact test for 2 by 2 tables of "on LLT combination versus not".

†Within 6 months before or at index visit.

‡For initial 6-month post-index follow-up period.

Table S4. Multivariable binary logistic regression analyses for post-index* active drug prescriptions

	Odds Ratio (95% CI)			
	**Any statin**	**High-intensity statin**	**Ezetimibe**	**PCSK9 inhibitor**
Cohort				
Passive alert	Reference	Reference	Reference	Reference
Active alert	0.13 (0.07–0.21)**	0.24 (0.16–0.37)**	4.69 (3.22–6.96)**	4.07 (1.92–9.46)**
Age				
Per year increase	0.99 (0.97–1.00)	0.98 (0.97–1.00)	1.00 (0.98–1.02)	0.99 (0.96–1.02)
Sex				
Male	Reference	Reference	Reference	Reference
Female	0.88 (0.57–1.38)	0.67 (0.46–0.96)**	0.63 (0.42–0.93)**	1.22 (0.58–2.46)
Race				
Black or African American	1.11 (0.43–2.99)	1.19 (0.53–2.67)	0.55 (0.22–1.19)	Not in the model
White or Caucasian	Reference	Reference	Reference	Not in the model
All others	0.82 (0.43–1.58)	1.07 (0.61–1.85)	1.33 (0.80–2.18)	Not in the model
Ethnicity				
Non-Hispanic or Latino	Reference	Reference	Reference	Not in the model
Hispanic or Latino	1.68 (0.40–6.87)	1.78 (0.44–6.90)	0.73 (0.18–2.26)	Not in the model
BMI				
Per unit increase	1.01 (0.98–1.05)	1.01 (0.98–1.04)	1.02 (0.99–1.05)	1.02 (0.96–1.08)
Comorbidities				
Hypertension	1.05 (0.66–1.67)	1.10 (0.75–1.62)	0.84 (0.56–1.27)	0.89 (0.41–2.06)
Diabetes	0.87 (0.53–1.42)	0.76 (0.51–1.12)	1.25 (0.83–1.88)	1.60 (0.74–3.32)
Smoking status				
Current smoker	Reference	Reference	Reference	Not in the model
Former smoker	0.66 (0.30–1.37)	1.11 (0.62–2.00)	1.45 (0.80–2.72)	Not in the model
Never smoked	0.88 (0.41–1.82)	1.30 (0.72–2.33)	0.95 (0.52–1.81)	Not in the model
Pre-index LDL-C†				
Missing	Reference	Reference	Reference	Reference
70–99 mg/dL	0.93 (0.54–1.62)	0.76 (0.49–1.16)	1.61 (1.01–2.58)**	1.08 (0.37–3.20)
≥100 mg/dL	0.78 (0.49–1.23)	0.97 (0.66–1.44)	2.86 (1.83–4.54)**	3.57 (1.53–9.37)**
Pre-index LLT therapeutic class†				
Not the same class	Reference	Reference	Reference	Reference
Same class	174.2 (90.7–363.6)**	215.9 (111.3–466.8)**	88.6 (43.0–204.4)**	359.5 (61.8–6923)**

BMI, body-mass index; CI, confidence interval; LDL-C, low-density lipoprotein cholesterol; LLT, lipid-lowering therapy; PCSK9, proprotein convertase subtilisin/kexin type 9.

** $P < .05$.

*For initial 6-month post-index follow-up period.

†Recorded within 6 months before or on index visit.

Table S5. Post-index last LDL-C measure at the end of each follow-up period

	**Passive-alert cohort (n = 733)**	**Active-alert cohort (n = 587)**	***P* value***
0–6 months post-index			
Missing, n (%)	365 (49.8)	237 (40.4)	<.001
Median (IQR), mg/dL	64.5 (51.0–84.3)	68.0 (56.0–86.0)	.086
0–12 months post-index			
Missing, n (%)	232 (31.7)	149 (25.4)	.015
Median (IQR), mg/dL	66.0 (52.0–84.0)	69.0 (55.0–86.0)	.029
0–18 months post-index			
Missing, n (%)	181 (24.7)	124 (21.1)	.144
Median (IQR), mg/dL	64.0 (51.0–83.0)	69.0 (54.0–89.0)	.006
0–24 months post-index			
Missing, n (%)	169 (23.1)	115 (19.6)	.146
Median (IQR), mg/dL	64.0 (51.0–83.0)	69.0 (52.0–90.0)	.016

IQR, interquartile range; LDL-C, low-density lipoprotein cholesterol.

*Chi-square when comparing frequencies and Mann-Whitney when comparing medians.

Table S6. Multivariable analysis of LDL-C monitoring and goal attainment

	Hazard Ratio (95% CI)		
	Time to LDL-C measure	Time to LDL-C ≤70 mg/dL*	Time to LDL-C ≤55 mg/dL*
Cohort			
Passive alert	Reference	Reference	Reference
Active alert	1.15 (1.01–1.31)**	0.99 (0.84–1.16)	0.83 (0.67–1.03)
Age			
Per year increase	1.00 (0.99–1.01)	0.99 (0.99–1.00)	1.00 (0.99–1.01)
Sex			
Male	Reference	Reference	Reference
Female	0.98 (0.86–1.12)	0.72 (0.61–0.86)***	0.64 (0.51–0.80)***
Race			
Black or African American	0.74 (0.61–0.90)***	0.69 (0.53–0.90)***	0.70 (0.48–1.01)
White or Caucasian	Reference	Reference	Reference
All others	0.99 (0.76–1.30)	0.92 (0.67–1.28)	1.40 (0.96–2.05)
Ethnicity			
Non-Hispanic or Latino	Reference	Reference	Reference
Hispanic or Latino	1.10 (0.71–1.72)	1.59 (0.97–2.60)	1.99 (1.12–3.51)**
BMI			
Per unit increase	1.00 (0.99–1.01)	0.98 (0.97–0.99)***	0.98 (0.96–0.99)***
Comorbidities			
Hypertension	1.08 (0.93–1.25)	1.08 (0.90–1.29)	1.05 (0.83–1.33)
Diabetes	1.06 (0.92–1.23)	1.09 (0.92–1.30)	1.26 (1.01–1.58)**
Smoking status			
Current smoker	Reference	Reference	Reference
Former smoker	1.28 (1.03–1.60)**	1.18 (0.89–1.57)	1.00 (0.69–1.46)
Never smoked	1.25 (0.99–1.56)	1.36 (1.03–1.81)**	1.26 (0.87–1.83)
Pre-index LDL-C†			
Missing	Reference	Reference	Reference
70–99 mg/dL versus missing (reference)	1.07 (0.92–1.25)	0.78 (0.66–0.94)***	0.59 (0.47–0.75)***
≥100 mg/dL versus missing (reference)	1.24 (1.06–1.45)***	0.64 (0.53–0.78)***	0.50 (0.39–0.64)***
Post-index LLT type (0–6 months)			
High-intensity statin versus high-intensity statin not prescribed (reference)	1.17 (1.02–1.34)**	1.56 (1.30–1.86)***	1.69 (1.32–2.17)**
Ezetimibe versus ezetimibe not prescribed (reference)	1.09 (0.91–1.31)	0.85 (0.67–1.06)	1.08 (0.81–1.45)
PCSK9 inhibitor versus PCSK9 inhibitor not prescribed (reference)	1.56 (1.10–2.22)**	0.83 (0.52–1.32)	1.52 (0.89–2.61)

BMI, body-mass index; CI, confidence interval; LDL-C, low-density lipoprotein cholesterol; LLT, lipid-lowering therapy; PCSK9, proprotein convertase subtilisin/kexin type 9.

** $P < .05$; *** $P < .01$.

[*]For patients with values.

[†]Recorded within 6 months before or on index visit.

Table S7. Physician evaluation of the active best-practice alert prompt (n = 88)

Questions and Responses	n (%)
Do you feel your training was effective to understand the protocol and maneuver through the EHR prompts?	
Yes	84 (95)
No	4 (5)
On a scale of 1–4, how do you find maneuvering through the Amgen CCP protocol?	
1-Very easy	16 (18)
2-Somewhat easy	49 (56)
3-Somewhat difficult	20 (23)
4-Very difficult	3 (3)
On a scale of 1–4, how effective do you believe the quality initiative has been in improving lipid management in your patients with a recent myocardial infarction?	
1-Very effective	20 (23)
2-Somewhat effective	51 (58)
3-Somewhat ineffective	6 (7)
4-Not at all effective	11 (13)
On a scale of 1–4, how do you find prescribing a PCSK9 inhibitor with the Amgen CCP protocol?	
1-Very easy	20 (23)
2-Somewhat easy	41 (47)
3-Somewhat difficult	25 (28)
4-Very difficult	2 (2)
How much did the EHR prompts influence your decision to prescribe lipid-lowering therapy?	
Never	8 (9)
Little	25 (28)
Somewhat	42 (48)
Often	13 (15)
How much extra time did the protocol add for you to complete your patient encounter?	
Less than 5 minutes	69 (78)
5 to 10 minutes	17 (19)
More than 10 minutes	2 (2)

CCP, Cardiology Consultants of Philadelphia; EHR, electronic health record; PCSK9, proprotein convertase subtilisin/kexin type 9.

Figure S1. Active best-practice alert prompts flow in EHR systems

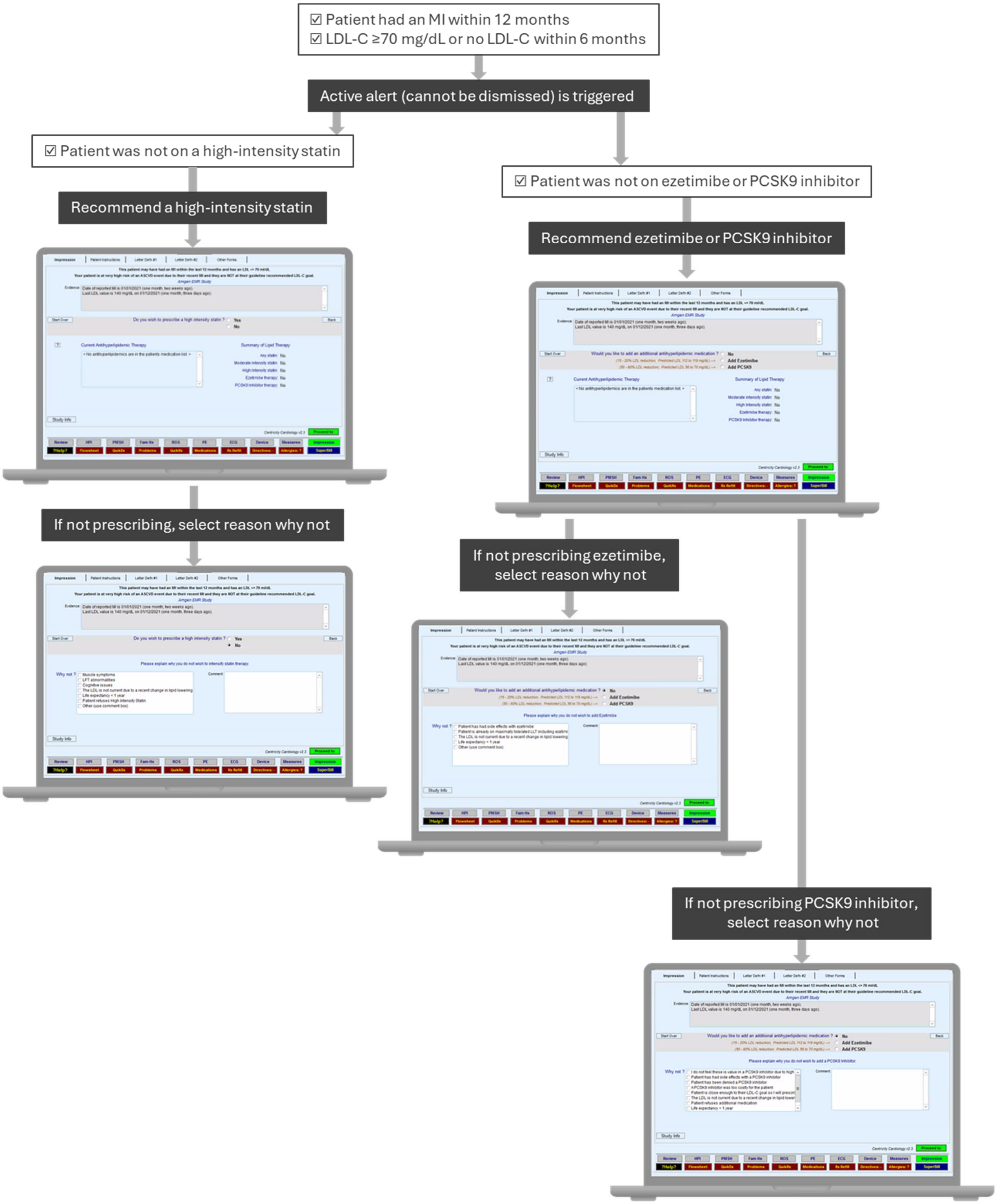

EHR, electronic health record; LDL-C, low-density lipoprotein cholesterol, MI, myocardial infarction; PCSK9, proprotein convertase subtilisin/kexin type 9.

Figure S2. Effect of intervention on number of post-index LLTs prescribed

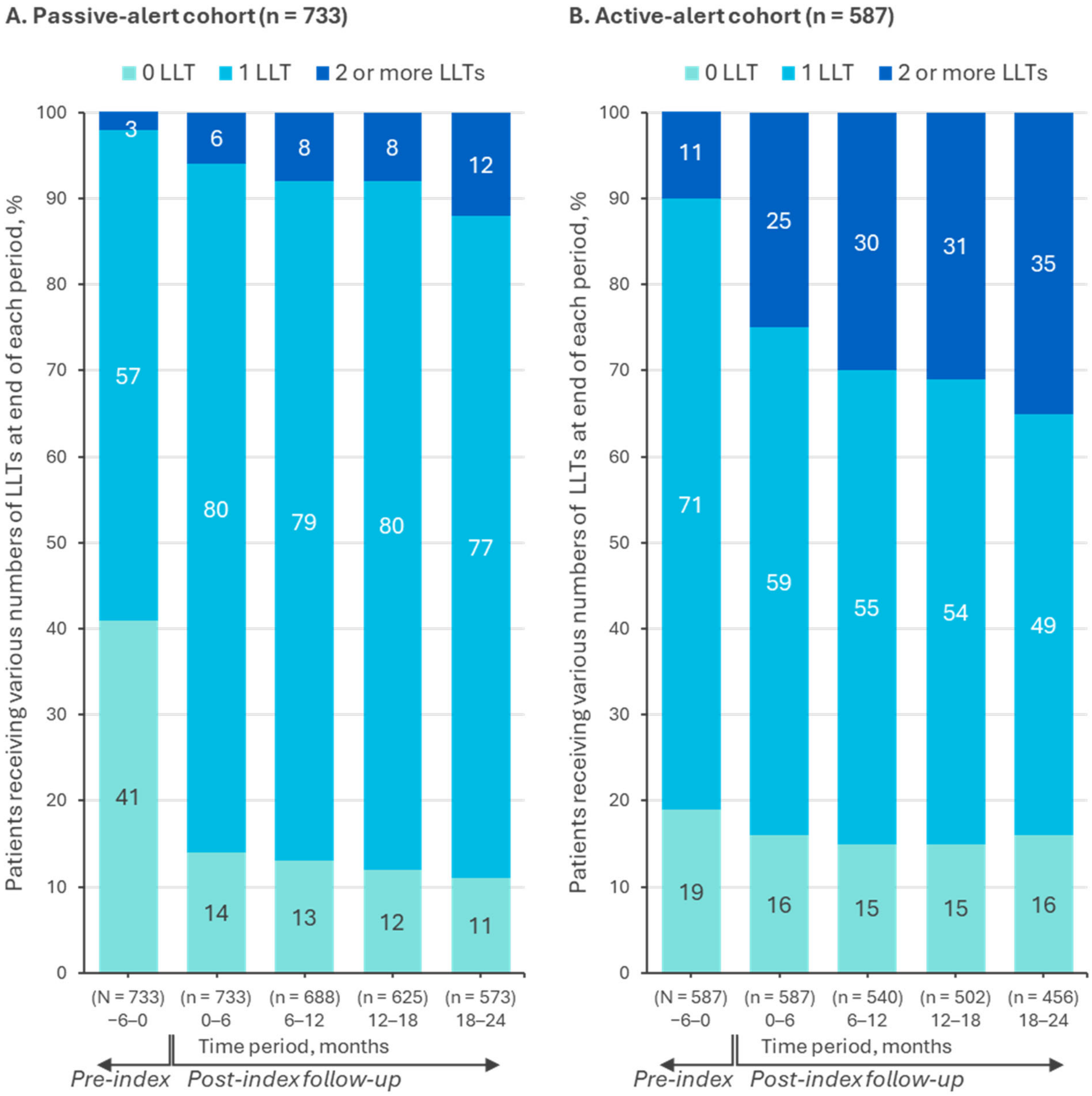

LLT, lipid-lowering therapy.

Figure S3. Effect of intervention on LLT combinations prescribed

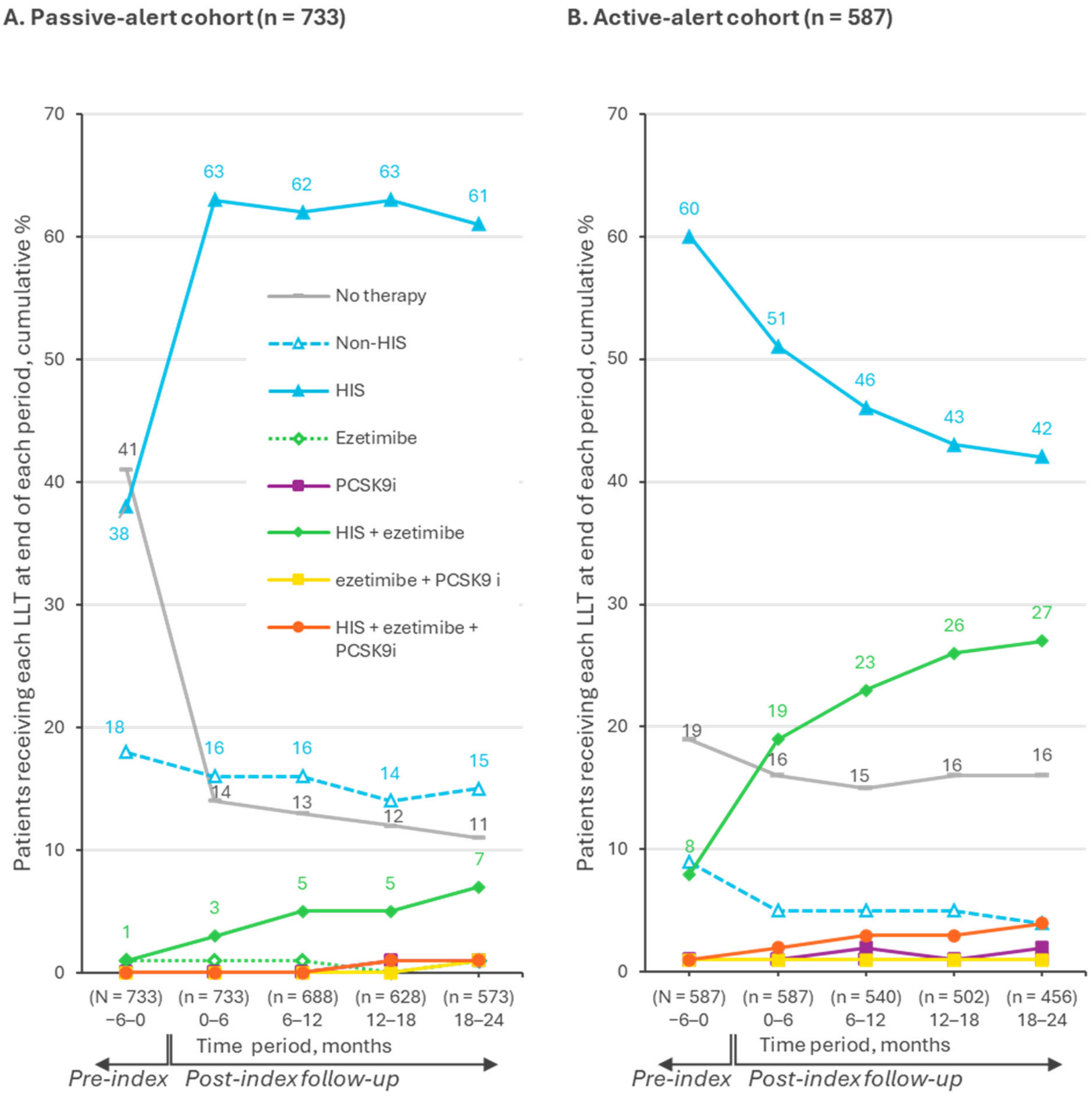

HIS, high-intensity statin; LLT, lipid-lowering therapy; PCSK9i, proprotein convertase subtilisin/kexin type 9 inhibitor.

Figure S4. Number of post-index LLTs prescribed by pre-index LDL-C level categories in each cohort

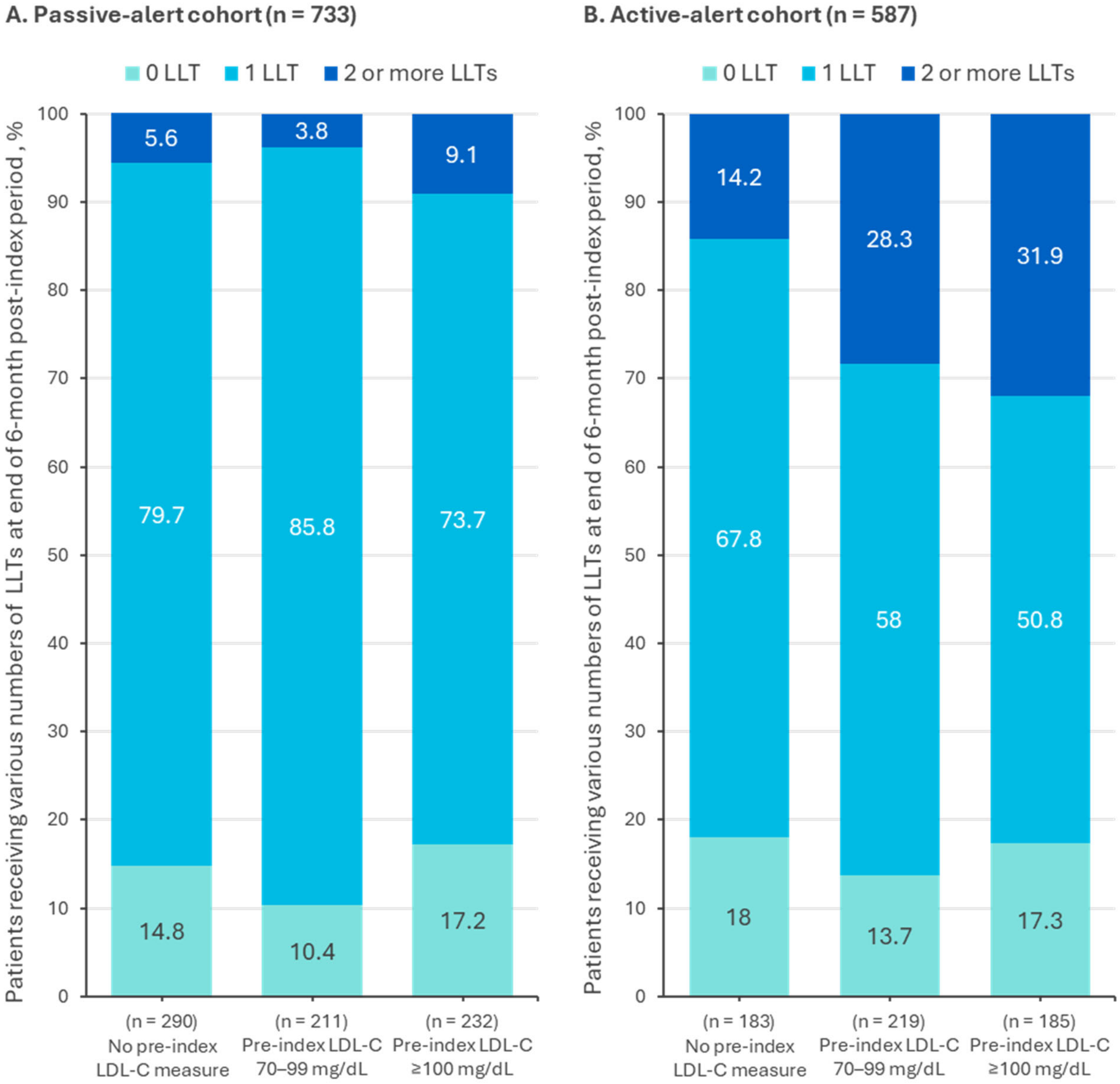

LDL-C, low-density lipoprotein cholesterol; LLT, lipid-lowering therapy.

Figure S5. Cumulative proportions of patients with unmeasured LDL-C or not at LDL-C goal.

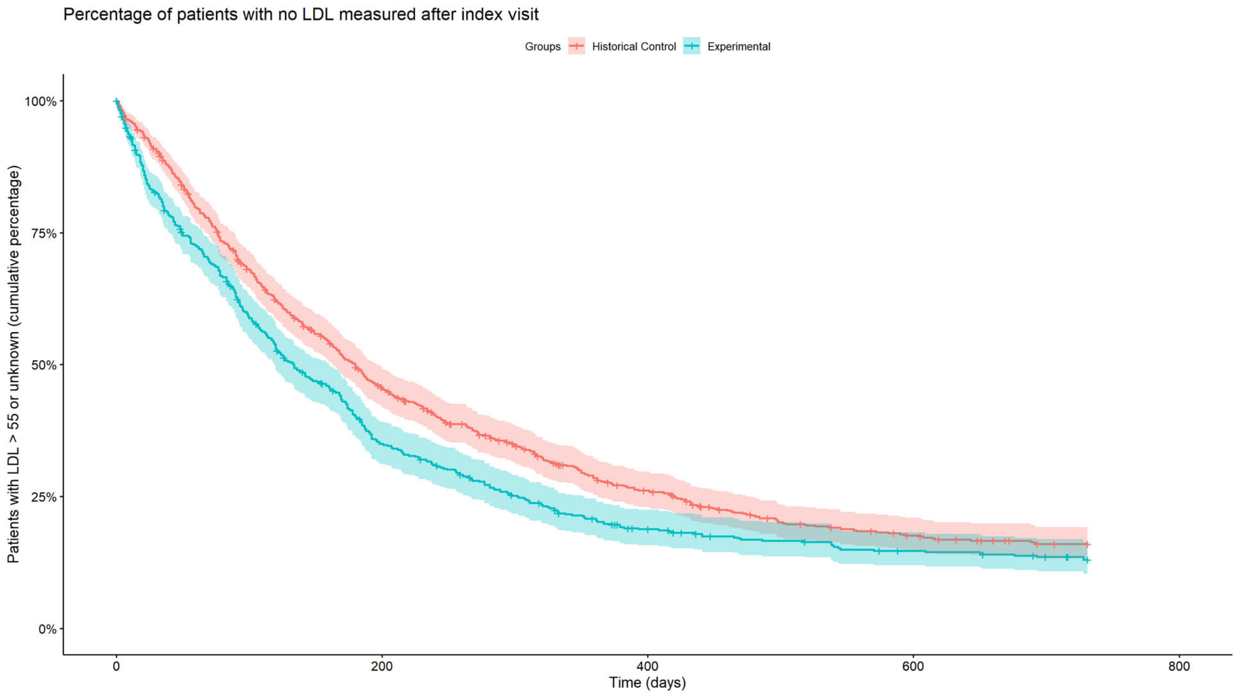

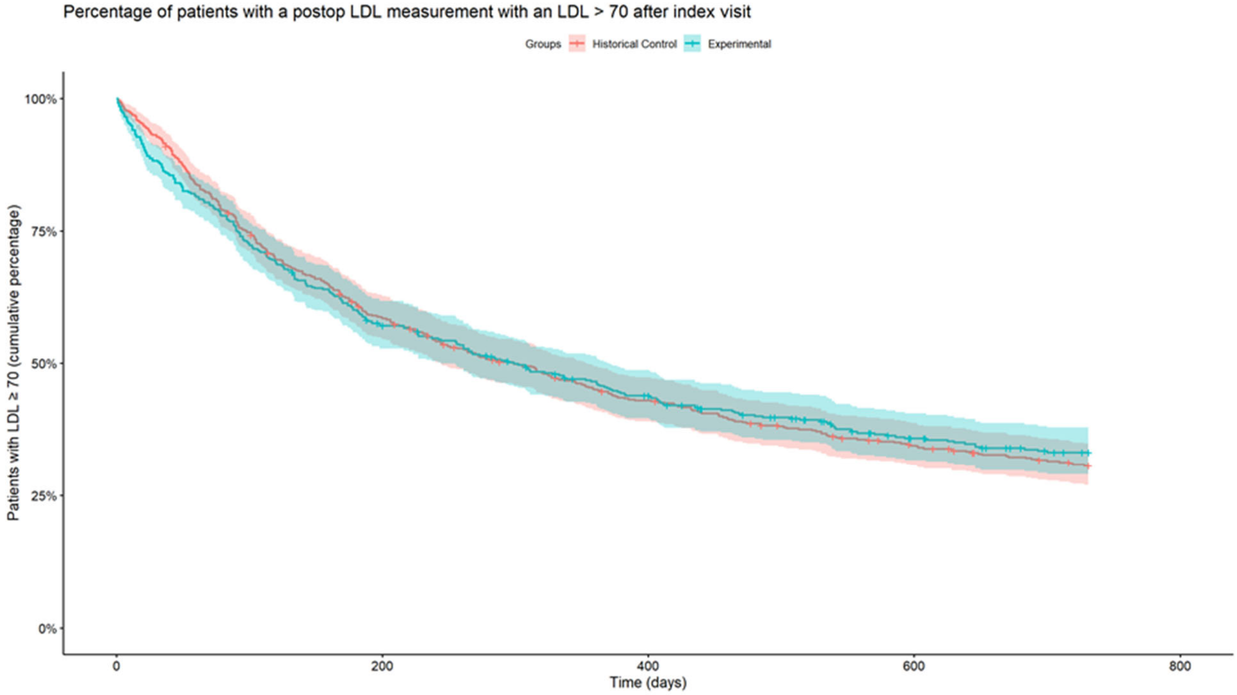

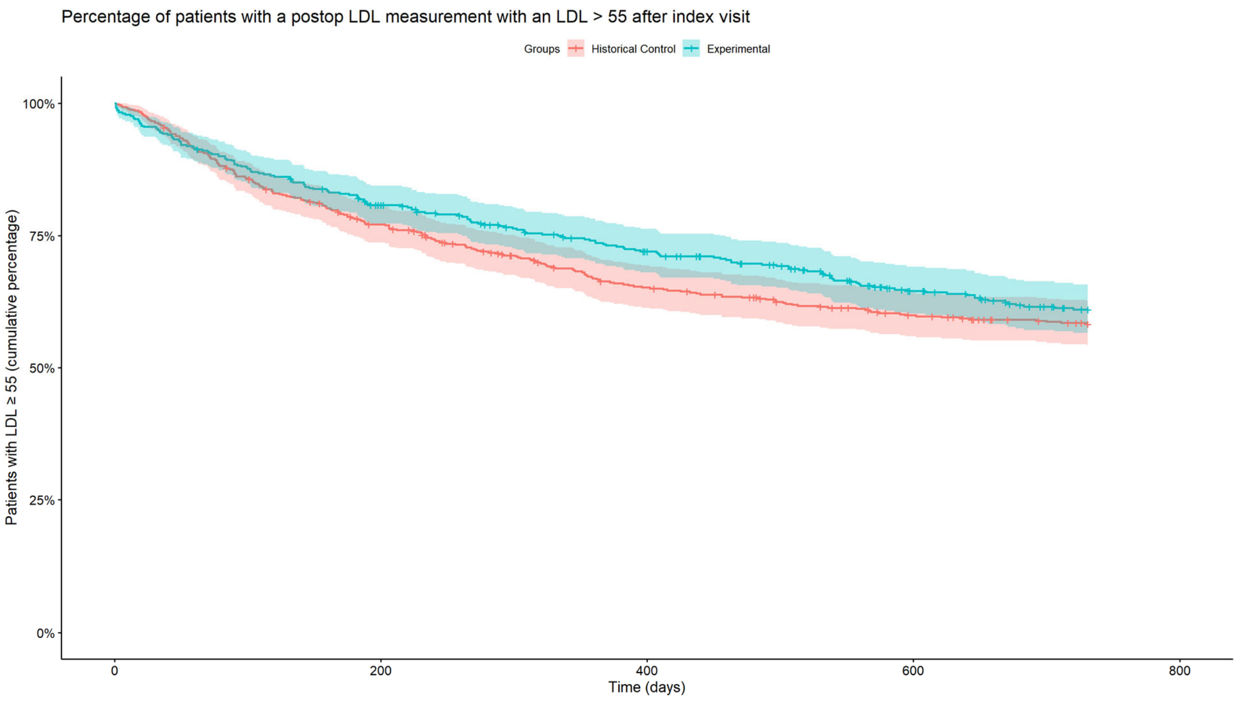

Shaded areas represent 95% confidence intervals.

LDL, low-density lipoprotein; LDL-C, low-density lipoprotein cholesterol.

Figure S6. Post-index LDL-C <70 mg/dL goal attainment by number of pre-index LLTs prescribed

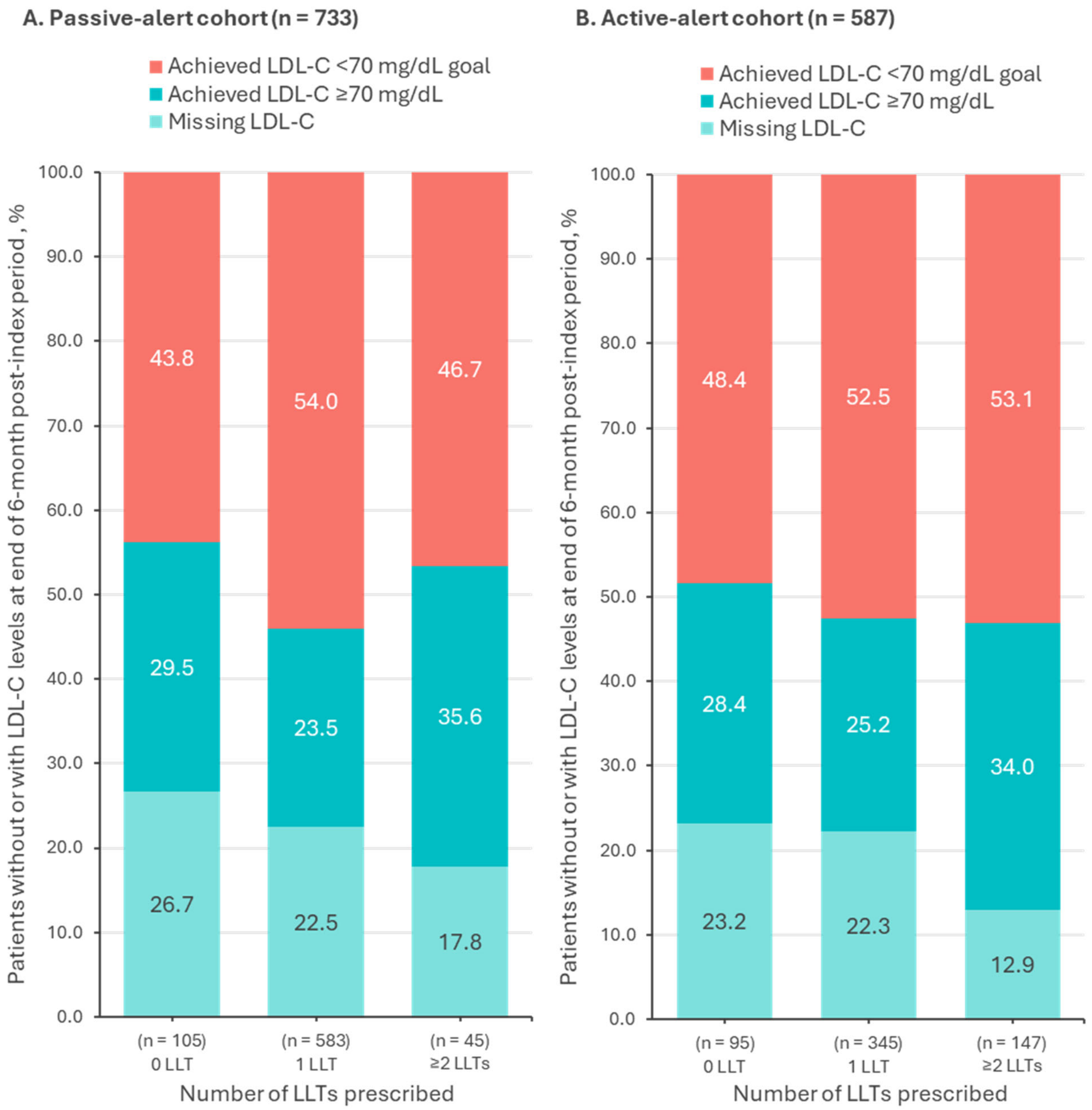

LDL-C, low-density lipoprotein cholesterol; LLT, lipid-lowering therapy.

Figure S7. Post-index LDL-C <55 mg/dL goal attainment by number of LLTs prescribed

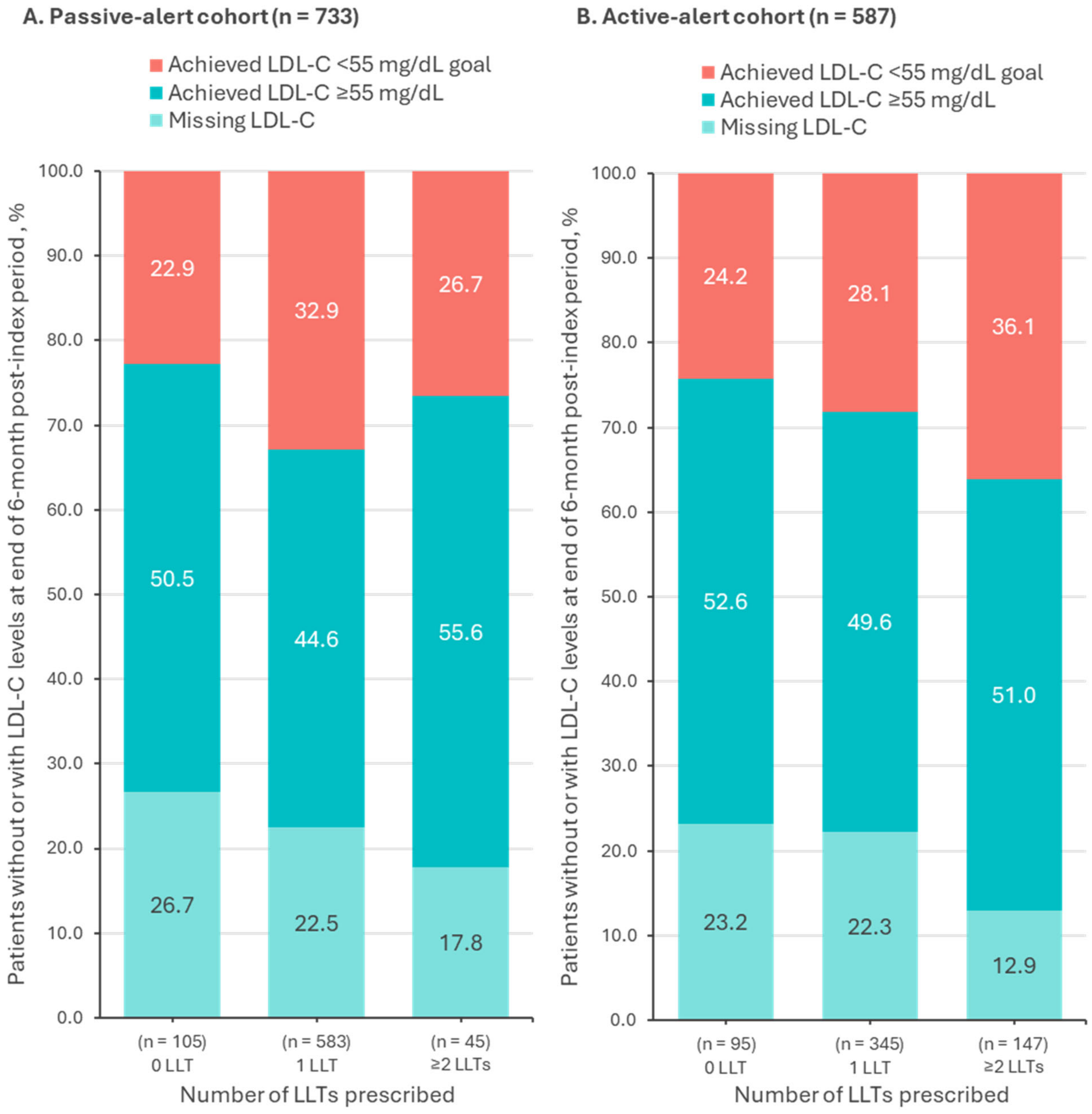

LDL-C, low-density lipoprotein cholesterol; LLT, lipid-lowering therapy.
