# Supplementary material for: Electronic Health Record Alerts to Improve Lipid Lowering After a Recent Myocardial Infarction
Source: J Am Heart Assoc. 2026 Jun 9;15(12):e047116. doi: 10.1161/JAHA.125.047116 (PMC13323587; doi:10.1161/JAHA.125.047116)
Supplement: Supplementary file 2 — STROBE Checklist [file JAH3-15-e047116-s002.pdf]

STROBE Statement—checklist of items that should be included in reports of observational studies

	Item No	Recommendation	Met	Relevant sections/text in manuscript
**Title and abstract**	1	(*a*) Indicate the study's design with a commonly used term in the title or the abstract	Yes	The study design—an observational, quality initiative, with sequential retrospective and prospective cohorts—is specified in the "Background" and "Methods" sections of the abstract (on page 3 of the manuscript)
		(*b*) Provide in the abstract an informative and balanced summary of what was done and what was found	Yes	This information is provided in the "Methods" and "Results" sections of the abstract (page 3)
**Introduction**				
Background/rationale	2	Explain the scientific background and rationale for the investigation being reported	Yes	The scientific background and the literature about the rationale to implement strategies to increase sub-optimal LDL-C guideline-recommended management are summarized in the "Introduction" section (page 7)
Objectives	3	State specific objectives, including any prespecified hypotheses	Yes	This information appears in the last 2 sentences of the "Introduction" section (pages 7-8).<br>Objective: *We evaluated the impact of changing from a passive to an active alert for patients with a recent MI who were not receiving 2018 guideline-recommended care.*[5]<br>Hypothesis: *Our hypothesis was that the active alert would improve adoption of the 2018 management of cholesterol guideline recommendations by measuring LDL-C and prescribing combination LLTs more often.*
**Methods**				
Study design	4	Present key elements of study design early in the paper	Yes	This information is provided in the second sentence of the "Study design" section (page 9)
Setting	5	Describe the setting, locations, and relevant dates, including periods of recruitment, exposure, follow-up, and data collection	Yes	Setting and location (Cardiology Consultants of Philadelphia [CCP]) are described in the first sentence of the "Study design" section (pages 9-10)<br>Relevant dates are provided in the "Study design" and "Participants" sections (data collection: February 2017 through October 2022; active-alert cohort recruitment: August 2020–January 2022; passive-alert cohort recruitment: February 2018–July 2019; follow-up: 24 months) (pages 9-10)

Participants	6	(*a*) *Cohort study*—Give the eligibility criteria, and the sources and methods of selection of participants. Describe methods of follow-up	Yes	Eligibility criteria (very high cardiovascular risk defined by elevated or missing LDL-C within 6 months of index visit and recent MI within 12 months of index visit) are described in the "Participants" section of the Methods (pages 9-10)<br>Data source (CCP cardiac catheterization records, EHR system, and physician survey) and collection, as well as methods of follow-up are specified in the "Study design", "Participants", and "EHR Alert" sections of the Methods (pages 9-10)
Variables	7	Clearly define all outcomes, exposures, predictors, potential confounders, and effect modifiers. Give diagnostic criteria, if applicable	Yes	Outcomes are defined in the "Outcomes" section and include prescribed LLTs, present/missing LDL-C tests, and LDL-C levels categorized as above/below guideline-specified goals (70 mg/dL and 55 mg/dL), as well as physician-reported reasons for not prescribing LLTs (page 11)<br>Exposures (passive and active alerts) are defined in the "EHR Alert" section (page 10)<br>Relevant diagnostic criteria are provided in Table S1 of the Supplement
Data sources/ measurement	8*	For each variable of interest, give sources of data and details of methods of assessment (measurement). Describe comparability of assessment methods if there is more than one group	Yes	The source of data is the same for baseline variables and outcomes, which are all taken from the CCP EHR system, as described in the "Participants", "Baseline variables", and "Outcomes" sections of the Methods (pages 9-11)
Bias	9	Describe any efforts to address potential sources of bias	Partly	This is an implementation science study, which is an exploratory, quality initiative, testing the feasibility and effectiveness of implementation strategies in a real-world clinical practice
Study size	10	Explain how the study size was arrived at	Yes	This was determined by the study periods and participant inclusion criteria, which are described in the "Study design" and "Participants" sections of the Methods (pages 9-10)
Quantitative variables	11	Explain how quantitative variables were handled in the analyses. If applicable, describe which groupings were chosen and why	Yes	Handling of quantitative variables is described in the "Analyses" section of the Methods (page 12)
Statistical methods	12	(*a*) Describe all statistical methods, including those used to control for confounding	Yes	The "Analyses" section of the Methods describes all applied statistical tests and software tools used (page 12)
		(*b*) Describe any methods used to examine subgroups and interactions	N/A	Not applicable
		(*c*) Explain how missing data were addressed	No covered	Where a data point was genuinely missing (without being part of group selection criteria, such as pre-index visit LDL) in lab

				values, vital statistics, or text entry of a value not able to be recoded to a numeric value, a categorical variable with an explicit "missing" level was substituted for the original numeric value in modeling.
		(*d*) *Cohort study*—If applicable, explain how loss to follow-up was addressed	Not covered	Patient loss to follow-up was addressed in long-term or time-to-event analyses by censoring cases by the last date associated with an encounter, lab value, or other observation in the patient EHR system.<br>For periodic analyses, a patient was considered "followed" into a time period if their last observed date was beyond the start of a time period (i.e., a patient with a last observed date at 6 months and 10 days post-index would be considered observed into the 6–12 month time period).
		(*e*) Describe any sensitivity analyses	N/A	Post-regression E-values were calculated to assess the relative strength of an unobserved confounder or confounders associated with the risk and outcome required to reduce an observed relationship to a null value.
**Results**				
Participants	13*	(*a*) Report numbers of individuals at each stage of study—e.g., numbers potentially eligible, examined for eligibility, confirmed eligible, included in the study, completing follow-up, and analyzed	Yes	Numbers and percentages of patients who triggered each alert are reported in the first sentence of the "Baseline characteristics" section of the Results (page 13)
		(*b*) Give reasons for non-participation at each stage	N/A	Not applicable
		(*c*) Consider use of a flow diagram	N/A	Not applicable
Descriptive data	14*	(*a*) Give characteristics of study participants (e.g., demographic, clinical, social) and information on exposures and potential confounders	Yes	Baseline or pre-index characteristics of patients in each cohort are described in Table 1 and are summarized in the "Baseline characteristics" section of the Results (page 13)
		(*b*) Indicate number of participants with missing data for each variable of interest	Partly	Patients with missing race are included in "other" category
		(c) Cohort study—Summarize follow-up time (e.g., average and total amount)	N/A	Not applicable
Outcome data	15*	*Cohort study*—Report numbers of outcome events or summary measures over time	Yes	Percentages of patients receiving different LLTs over time are displayed in Figure 1<br>Cumulative percentages of patients with measured LDL-C and attaining LDL-C goals of <70 mg/dL or <55 mg/dL over time are displayed in Figure 2

				Physician-reported reasons for active alert dismissal are displayed in Figure 4
Main results	16	(*a*) Give unadjusted estimates and, if applicable, confounder-adjusted estimates and their precision (e.g., 95% confidence interval). Make clear which confounders were adjusted for and why they were included	Partly	All variables included in models are reported in text/tables/figures, with 95% confidence intervals. These are reported as adjusted values only. Unadjusted model coefficients were calculated as part of model building but are not included in the paper for brevity.
		(*b*) Report category boundaries when continuous variables were categorized	N/A	Variance of numeric variables are reported as median with 25th and 75th percentiles when reported. The full value range is not reported.
		(*c*) If relevant, consider translating estimates of relative risk into absolute risk for a meaningful time period	N/A	Odds ratios are paired with the actual increase in magnitude between groups where possible.
Other analyses	17	Report other analyses done—e.g., analyses of subgroups and interactions, and sensitivity analyses	Partly	Post-regression E-values were calculated to assess the relative strength of an unobserved confounder or confounders associated with the risk and outcome required to reduce an observed relationship to a null value.
**Discussion**				
Key results	18	Summarize key results with reference to study objectives	Yes	Key results are summarized at the beginning of the Discussion section (page 17)
Limitations	19	Discuss limitations of the study, taking into account sources of potential bias or imprecision. Discuss both direction and magnitude of any potential bias	Yes	An extensive discussion of the limitations of our study appears in the "Limitations" section of the Discussion (pages 20-21)
Interpretation	20	Give a cautious overall interpretation of results considering objectives, limitations, multiplicity of analyses, results from similar studies, and other relevant evidence	Yes	The "cautious" interpretation of the study findings (without implications of causality) in comparison with previously published literature is described throughout the "Discussion" (pages 17-20) and further summarized in the "Conclusions" (page 22)
Generalizability	21	Discuss the generalizability (external validity) of the study results	Yes	The limitations for the generalizability of the findings is described in the "Limitations" section of the Discussion (pages 20-21)
**Other information**				
Funding	22	Give the source of funding and the role of the funders for the present study and, if applicable, for the original study on which the present article is based	Yes	The funding source is stated in the "Acknowledgements" section (page 24)

*Give information separately for cases and controls in case-control studies and, if applicable, for exposed and unexposed groups in cohort and cross-sectional studies.

Note: An Explanation and Elaboration article discusses each checklist item and gives methodological background and published examples of transparent reporting. The STROBE checklist is best used in conjunction with this article (freely available on the Web sites of PLoS Medicine at http://www.plosmedicine.org/, Annals of Internal Medicine at http://www.annals.org/, and Epidemiology at http://www.epidem.com/). Information on the STROBE Initiative is available at www.strobe-statement.org.
